# Assessing Leaching of Potentially Hazardous Elements from Cookware during Cooking: A Serious Public Health Concern

**DOI:** 10.3390/toxics11070640

**Published:** 2023-07-24

**Authors:** Saif Ali Ali Sultan, Fawad Ahmed Khan, Abdul Wahab, Batool Fatima, Hira Khalid, Ali Bahader, Sher Zaman Safi, Chandrabose Selvaraj, Abid Ali, Suliman Yousef Alomar, Muhammad Imran

**Affiliations:** 1 Biochemistry Section, Institute of Chemical Sciences, University of Peshawar, Peshawar 25120, Pakistan; 2 Key Laboratory of Applied Surface and Colloid Chemistry, Ministry of Education, School of Chemistry & Chemical Engineering, Shaanxi Normal University, Xi’an 710062, China; aaabdulwahab0@gmail.com; 3 Department of Biochemistry, Bahauddin Zakariya University, Multan 60800, Pakistan; 4 Tianjin Key Laboratory of Molecular Optoelectronic Sciences, Department of Chemistry, School of Science, Tianjin University & Collaborative Innovation Center of Chemical Science and Engineering, Tianjin 300072, China; 5 Department of Chemistry, Hazara University, Mansehra 21120, Pakistan; 6 Faculty of Medicine, Bioscience and Nursing, MAHSA University, Bandar Saujana Putra, Jenjarom 42610, Malaysia; dr.szsafi@gmail.com; 7 IRCBM, COMSATS University Islamabad Lahore Campus, Punjab 54000, Pakistan; 8 Center for Transdisciplinary Research, Department of Pharmacology, Saveetha Dental College and Hospitals, Saveetha Institute of Medical and Technical Sciences (SIMATS), Saveetha University, Chennai 600077, India; 9 Department of Zoology, Abdul Wali Khan University Mardan, Mardan 23200, Pakistan; 10 Zoology Department, College of Science, King Saud University, Riyadh 11451, Saudi Arabia

**Keywords:** cookware, hazardous metals, biomonitoring, food safety, human exposure

## Abstract

The intake of toxic metals from cooking utensils through food is of growing concern to the medical community. This intake poses serious risk to human health. In many developing countries, different types of contaminated metals scraps are used to make cooking utensils. The leaching of both nutritionally essential and toxic metals in significant quantities from cookware during the cooking process results in food contamination and poses a substantial health risk. In the present study, the leaching of some toxic and potentially toxic metals from cooking utensils into different solutions and food was investigated. A preliminary survey indicated that the majority of individuals tend to use aluminum cookware due to its affordability, overlooking the potential health risks associated with these inexpensive and lower-quality cooking utensils. XRF analysis revealed that aluminum, steel, and copper cookware had K, Ca, Pb, Cd, Ni, V, Sn Mo, Zn, Bi, and Tb as contaminants. In addition, aluminum (3.2 ± 0.25 to 4.64 ± 0.20 g/kg) and copper cookware (2.90 ± 0.12 g/kg) were highly contaminated with lead. The time and pH-dependent study revealed that leaching of metals (Al, Pb, Ni, Cr, Cd, Cu, and Fe, etc.) into food was predominantly from anodized and non-anodized aluminum cookware. More metal leaching was observed from new aluminum cookware compared to old. Acidic food was found to cause more metals to leach during cooking. Blood metal analysis of the local population revealed the presence of high concentrations of Al, Pb, Cd, and Ni. In conclusion, leaching of toxic or potentially toxic metals from cookware into food, especially from anodized and non-anodized aluminum cookware, poses a potential public health risk. Practical applications: Cooking utensils are routinely used for the preparation of food. However, the harmful impact posed by these essential items is largely unknown. The current research briefly explains the toxic metals leaching from cookware in a pH-dependent manner and leaves a message to the public, especially in developing countries like Pakistan, regarding the type of cookware suitable for cooking purposes.

## 1. Introduction

Cookware is broadly used for food preparation around the globe. There are different types of cookware in terms of composition, including aluminum, copper, stainless steel, cast iron and enameled cast iron, etc. The most common type is aluminum cookware [1], which is the most extensively used kitchen staple for cooking around the globe. Its long-standing use as a utensil is due to its low cost and its ability to conduct heat effectively, making it an ideal choice for cookware [2]. Aluminum reacts with acidic food, causing the metal to leach into the food and thereby make its way into the body. To ensure the aluminum remains intact, the cookware must be subjected to an electrochemical process called anodization. This forms a much thicker layer of non-reactive aluminum oxide, creating what is known as anodized or hard-anodized aluminum. As a result, most aluminum cookware is either coated with a non-stick layer or is anodized [3].

Stainless steel is utilized domestically and commercially for food preparation. However, it is more expensive than aluminum cookware. Steel cookware used for food preparation contains 18% Cr, 8% Ni, and 70 to 73% Fe. Steel cookware is also attacked by organic acids, especially at cooking temperatures. As such, Fe, Cr, and Ni discharge from the material into the food. However, they are comparatively more thermostable than aluminum cookware and can withstand sweltering temperatures [4]. The use of copper cookware is less common in homes, but it is broadly used in business cookware for food preparation. Copper cookware is more expensive than aluminum cookware. Copper cookware contains Cu, Ni, Sn, Fe, and Ag and is coated with Ni, Ag, Sn, and stainless steel to prevent food from encountering copper, as the ingestion of large amounts of copper can create health problems, including vomiting, nausea, kidney disease, and diarrhea [5].

The human body is composed of many chemical elements, including iron, iodine, zinc, copper, manganese, chromium, chloride, etc. These elements are essential for human life [6]. Some of the heavy elements have important physiological functions but can also cause serious cellular toxicity. Heavy metals, including Fe, Cr, Cu, etc., have deleterious effects on the human body when taken in more than the required and recommended levels [7]. Other elements, including Al, Cd, Ni, Pb, and As, have not yet been recognized for their physiological functions and the body obtains no benefits from them when they are inhaled, ingested through food, or absorbed through the skin. These metals have serious health consequences when they enter the human body through food intake [8].

There are many risk factors causing metal toxicity in humans, including exposure to wastewater, industrial effluents, vehicular emissions, etc. [8]. In the current study, we hypothesize various types of cookware as potential sources of metal toxicity in the Pakistani population, as the majority of the cookware used in Pakistan is made from metal scraps and is poorly anodized. Leaching of both nutritionally essential and toxic metals in high amounts during cooking leads to food contamination and hence poses a serious health risk to society [9]. Until now, no data from Pakistan have been reported regarding the health hazards of such cookware. This study was therefore designed to investigate the potential health risk posed by cookware manufactured in Pakistan.

## 2. Materials and Methods

### 2.1. Questionnaire Survey

A questionnaire survey was conducted to collect information about cookware, including the type of cookware used, the reason for selection of a specific type of cookware, knowledge about the associated hazard of a particular type of cookware, and knowledge about metals’ toxicity, etc. The survey was conducted in houses (*n* = 910), restaurants (*n* = 155), and shops (*n* = 54). All participants received a thorough explanation regarding the survey’s nature and objectives, and their consent, with full information, was obtained on a predesigned proforma.

### 2.2. Cookware Collection

Copper, stainless steel, and aluminum cookware were purchased from various cities (Peshawar, Nowshehra, Kohat, Abbottabadd, and Rawalpindi) in Pakistan. All types of cookware were locally made and were distributed throughout the country.

### 2.3. Determination of Elemental Composition of Cookware by X-ray Fluorescence (XRF)

The elemental composition of cookware was determined using XRF (EDX-7000, Na-U, Shimadzu, Kyoto, Japan) with a loose powder method, calibration with Al-Cu standard at Centralized Resource Laboratory University of Peshawar, Pakistan. An elemental analysis of cookware was performed to determine the composition and possible toxic metal contamination in cookware. Samples were cut into small pieces, polished, and analyzed via XRF equipped with an X-ray tube containing a Rhodium electrode, a high-performance silicon drift detector, and PCEDX-Navi software. The instrument was run at 50 kV and 1000 μA. At least three different regions of each sample collected from different areas were analyzed.

### 2.4. Determination of Metals Leaching in Food from Cookware

There is no standardized protocol for studying metals leaching from cookware. However, to assess metals leaching, acetic acid solution (4%), sodium bicarbonate solution (0.5 N), and deionized ultra-pure water were boiled in all cookware for 30, 60, and 120 min. The solutions were filtered and subjected to metals analysis using Atomic Absorption Spectrophotometer. Aluminum cookware is available both in anodized and non-anodized forms; hence, they were differentially examined for metals leaching. Because new and old cookware may have different thermal stabilities, both new and old samples were used in heat-mediated metal leaching. To investigate metals leaching from old cookware, different types of new cookware were purchased and used continuously for 2 months at least two times a day to cook various foods. Then, they were used to study metals leaching upon boiling solutions of acetic acid (4%), sodium bicarbonate (0.5 N), and ultra-pure deionized water. Samples were then analyzed using an atomic absorption spectrophotometer for Al, Ni, Pb, Cd, Cr, Cu, and Fe.

### 2.5. Determination of Metals Leaching from Aluminum Cookware during Cooking Meat

The study was further extended to cooking acidic foods in cookware and metals analysis was performed in food samples before and after cooking. About 250 g of meat was cut into small pieces and subjected to boiling in 500 mL of deionized ultra-pure water for 1 h in non-anodized and anodized aluminum cookware and then subjected to filtration (Solution A). For control, 250 g of meat was boiled in 500 mL of deionized ultra-pure water in a flask and then filtered (Solution B). Next, 5 mL of the filtrates was separately digested with nitric acid and hydrogen peroxide (2:1) mixture and analyzed using a atomic absorption spectrophotometer for Al, Pb, Cr, Cd, and Ni analysis. To remove the possible metal contamination by meat, the amount of metals determined in solution A was subtracted from solution B.

### 2.6. Analysis of Toxic Metals in Blood Samples

First, 2 mL blood was taken from well-fed normal individuals (*n* = 150) of both genders (age 15–50 years). In order to reduce the potential risk of exposure to toxic metals from other environmental sources, the study participants, consisting primarily of students and office workers, were carefully selected. The participants were chosen based on their residence in a relatively clean environment, which potentially resulted in lower exposure to other environmental pollutants. The collection of blood specimens was carried out by a certified technician (Phlebotomist) at Hyatabad Medical Complex Peshawar, Pakistan. The blood was mixed with 12 mL nitric acid and hydrogen peroxide (2:1) mixture in a 100 mL volumetric flask and kept for 10 min at room temperature followed by digestion for 2.5 h at 60–70 °C. After that, 8 mL of nitric acid and 6 drops of hydrogen peroxide were added to the flask and kept for 1.5 h at 60–70 °C. The mixture then was evaporated at 85–87 °C in a titration flask. After evaporation, 4 mL of nitric acid and three drops of hydrogen peroxide were added again to the flask and digested at 60–70 °C for 1 h. Again, the mixture was evaporated at 85–87 °C in a titration flask. After evaporation, the sample was cooled and filtered. The samples were analyzed using an atomic absorption spectrophotometer for metals analysis. Informed consent was given by all the subjects involved in this study. The study was approved by the University of Peshawar ethical board (No: 105/EC/F.LIFE/UOP-2017).

### 2.7. Statistical Analysis

All experiments were performed in triplicate and data were presented as Mean ± SD. *t*-test One-way ANOVA was used to calculate statistical differences. *p* ≤ 0.05 was regarded as statistically significant.

## 3. Results

### 3.1. Questionnaire Survey

**Figure 1:**
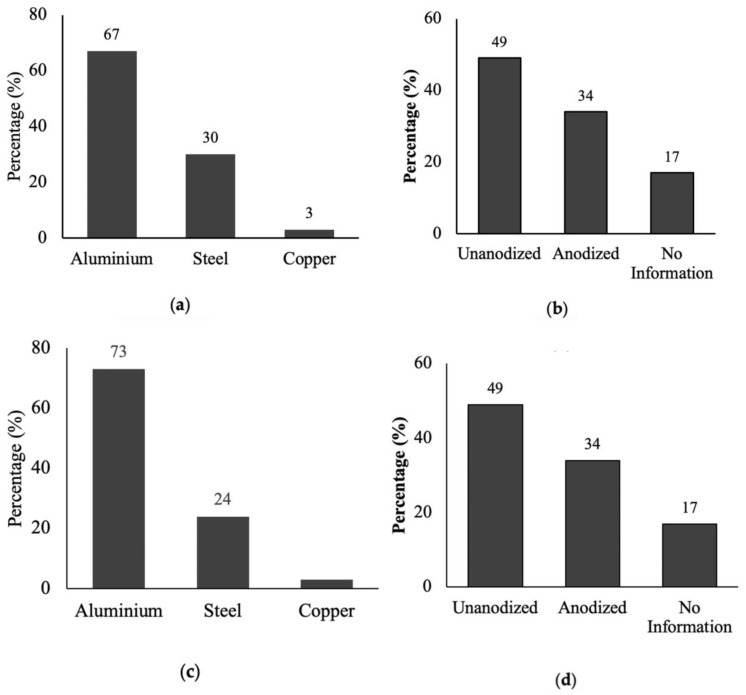
Types of cookware used in homes (**a**,**b**) and restaurants (**c**,**d**).

**Figure 2:**
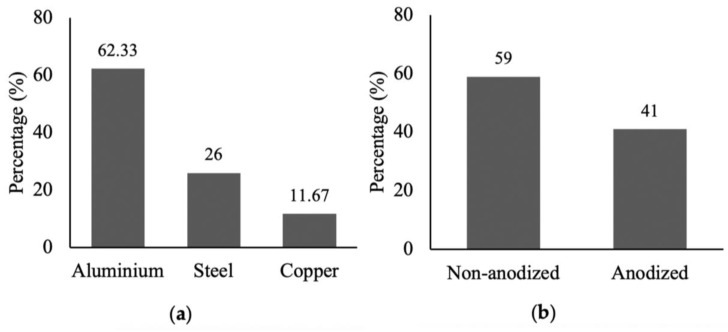
Types of cookware sold by shopkeepers, aluminium, steel, copper (**a**); anodized and non-anodized (**b**).

**Figure 3:**
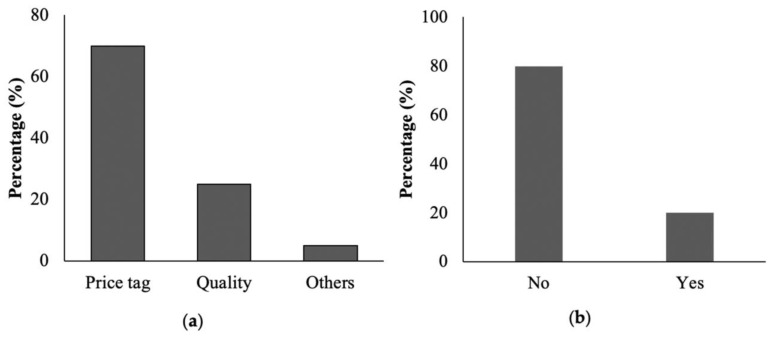
Reason for purchase of a type of cookware (**a**) and knowledge about the metals toxicity and other health hazard associated with the use of low-quality cookware (**b**).

To find out which type of cookware is used most widely by the local population, an extensive survey was conducted in cookware seller shops, homes, and restaurants. The gathered data indicate that both in restaurants and homes, aluminum cookware is more widely used compared to stainless steel and copper cookware (Figure 1). Furthermore, it was observed that non-anodized aluminum cookware (49%) is preferred over anodized aluminum cookware (34%) in homes, whereas in restaurants, anodized aluminum cookware (60%) was more popular than non-anodized (37%) aluminum cookware (Figure 1b,d). Interestingly, 17% of individuals at home and 3% of restaurant users were unable to distinguish between anodized and non-anodized aluminum cookware, respectively. These findings were reinforced through interviews with cookware shopkeepers (Figure 2). The survey conducted in local shops revealed that 62.33% of the sold cookware was aluminum, while stainless steel accounted for 26% and copper for 11.67% (Figure 2a). Among the aluminum cookware, non-anodized cookware (59%) had higher sales compared to anodized cookware (41%) (Figure 2b). The survey findings highlighted that the primary factor influencing cookware selection among the local community was affordability, with little consideration given to the quality of the cookware and the associated health risks (Figure 3).

### 3.2. Elemental Composition of Cookware by XRF

The elemental composition of new aluminum, stainless steel and copper cookware was determined via X-ray fluorometer (XRF). Several samples collected from different cities were analyzed for elemental composition. The data showed that the new cookware was not made up of pure Al, Fe, or Cu; instead, some impure raw materials were used to manufacture this cookware (Table 1). The new non-anodized aluminum cookware was found to contain Al, Fe, Pb, Cr, Ni, Cu, K, Zn, Ca, Mn, V, Bi, Tb, and Sn. The same elements were present in the new anodized aluminum cookware in different concentrations. On the other hand, the results of XRF analysis showed that elements present in new stainless-steel cookware were Fe, Cr, Mn, Ni, Cu, V, Bi, Se, and Ca (Table 1), while new copper cookware contained Cu, Zn, Si, Sn, Fe, Tb, Ca, Pb, I, Ni, S, and Mo in different concentrations. The XRF analysis showed that both new non-anodized and anodized aluminum cookware were highly contaminated with Pb, Cr, and Ni. Additionally, copper and stainless-steel cookware was contaminated with Ni, Sn, etc.

### 3.3. Metals Leaching in Food from New and Old Cookware

The effect of an acidic medium on metals leaching from aluminum, steel, and copper cookware was determined in a time-dependent manner (Table 2 and Table 3). Acetic acid solution (4%) was boiled in new cookware for 0.5, 1, and 2 h, and leached metals were analyzed using an atomic absorption spectrophotometer. The amount of metals leached from various cookware followed the order of non-anodized aluminum > anodized aluminum > steel > copper. Both non-anodized and anodized aluminum cookware showed high amounts of Al, Fe, Pb, and Cu leaching. The metal leaching showed a significant increase (*p* < 0.05) with an increase in boiling time. The metal leaching was also determined in old cookware at a 1 h time point. Interestingly, there was no significant difference (*p* < 0.05) between the new and old steel and copper cookware in terms of metals leaching in the acidic medium; however, the amount of metals leached from new non-anodized and anodized aluminum cookware was significantly (*p* > 0.05) higher than old aluminum cookware. Furthermore, old anodized aluminum cookware was more prone (*p* > 0.05) to metal leaching compared to new anodized cookware.

The effect of alkaline foods on metals leaching from various cookware was mimicked using sodium bicarbonate solution (0.5 M) time dependently (0.5, 1, and 2 h). The data obtained are presented in Table 4 and Table 5. The alkaline medium was also found to have a differential effect on metal leaching from various cookware. The metals leaching in an alkaline medium followed the same order as that of acetic acid; however, sodium bicarbonate had comparatively low metals leaching effect. A time-dependent increase in metal leaching was observed among all cookware. The old non-anodized aluminum cookware was more stable and was little affected by alkaline medium compared to new non-anodized aluminum cookware. Furthermore, the order of concentration in which metals leached under an alkaline medium was different than in an acidic medium for most of the metals. For example, Pb leaching in both non-anodized and anodized cookware in an alkaline medium was more than Fe, while in acidic medium, the Fe leaching was greater than that of Pb. Similarly, from steel cookware, more aluminum was leached than Ni under an alkaline medium, while from copper cookware, more Cr leaching was greater than Pb. This indicates the differential effect of pH on the amount and type of metals that leach from various cookware.

The effect of a neutral medium on metals leaching from various cookware was determined by boiling deionized ultra-pure water in time-dependent manner (0.5, 1, and 2 h). The data show that neutral medium has minimal effect on metals leaching from all cookware (Table 6 and Table 7) and is within the safe limits. Aluminum cookware showed higher metal leaching compared to steel and copper cookware. It is worth mentioning that the amount of metals leached both from new and old aluminum cookware was non-significantly (*p* > 0.05) different. The data reveal that neutral food causes less metal to leach from cookware compared to acidic and alkaline food (Table 8).

### 3.4. Metals Leaching from Aluminum Cookware during Cooking Meat

To further confirm metal leaching from aluminum cookware, both non-anodized and anodized aluminum cookware were subjected to cooking meat as a real acidic food sample. The meat was boiled in both non-anodized and anodized aluminum cookware for 1 h and the concentration of the selected metal was determined. Table 9 shows the concentration of Al, Pb, Cr, Cd, and Ni leached from new non-anodized and old anodized aluminum cookware. The results indicate that the amount of metals leached from new non-anodized aluminum cookware was significantly higher (*p* < 0.05) than that of anodized aluminum cookware. Moreover, old anodized aluminum cookware was more prone to metals leaching than new cookware, while in the case of non-anodized cookware, the new cookware showed significantly high (*p* < 0.05) metals leaching compared to old cookware.

### 3.5. Concentration of Toxic Metals in Blood Samples of Local Population

In order to investigate the potential ingestion of leached metals, the levels of serum metals were analyzed within the local population. Table 10 shows the average values of Al, Pb, Cd, and Ni in the blood serum of the local population. Metals analysis was performed for blood samples randomly collected from different individuals. It is worth mentioning that all these individuals were either students or people working in offices, and they were not directly exposed to contaminated environments. The concentrations of Al, Pd, Cd, and Ni in blood serum were 13.34 ± 0.49, 1.314 ± 0.19, 0.51 ± 0.016, and 2.17 ± 0.10 mg/L, respectively.

## 4. Discussion

The current research provides an overview of the health and environmental impacts of common cookware choices based on data gathered, studies compared, and all options dutifully considered. We examined the consumer usage conditions and toxic metals released from the cookware. Our study showed that aluminum cookware was more extensively used for cooking purposes compared to stainless steel and copper cookware both in homes and restaurants. More than half of the cookware used in homes was made of aluminum, mainly because of its low price and good thermal conductivity (Figure 3). However, it is worth mentioning that the use of non-anodized aluminum cookware in homes was more prevalent than anodized cookware, while most restaurants preferred to use anodized aluminum cookware because of its good quality.

XRF analysis showed that the locally produced cookware was not composed of pure raw material; rather, it included different toxic heavy metals, including radioactive metals. The composition of stainless steel and copper cookware showed that it was less contaminated with heavy metals, including Pb, Ni, Cd, etc., compared to non-anodized and anodized aluminum cookware and was comparatively safer to use for cooking purposes. A survey of the local industries revealed that in Pakistan, most of the cookware used in homes is locally manufactured from recycled scrap materials such as computer parts, automobile parts, electrical wires, cans, and other industrial debris. Most of these recycled materials are contaminated with various toxic elements, including Pb, As, Ni, Cd, Sn, Si, etc. All these elements have relatively high atomic densities, atomic weights, or atomic numbers and are referred to as heavy metals. Their contamination of various types of cookware poses a serious health risk to the local community. Moreover, these various types of cookware are either uncoated (not anodized) or poorly protected, which further increases metals leaching during food preparation. Thus, the composition and the extent of toxic metals leaching into the food during cooking are the main factors evaluating the healthfulness of cookware. The leakage of such toxic metals during cooking can hence lead to serious health problems, including cancer, cardiovascular, gastrointestinal, and severe respiratory disorders. A study by Weidenhamer et al. has reported contamination of aluminum cookware with some toxic metals, including Ni, Pb, and Cd, in many developing countries, including Bangladesh, India, and Vietnam [3].

The concentration of heavy metals leached from new non-anodized aluminum cookware was significantly (*p* < 0.05) higher than that of old non-anodized aluminum cookware, demonstrating that the outer rough layer is removed during cooking and non-anodized aluminum cookware becomes comparatively more resistant to metals leaching. However, anodized cookware becomes more sensitive to metals leaching upon repeated use. This shows that the anodized aluminum cookware transforms into non-anodized aluminum cookware after being used several times, as the anodized layer is constantly being leached out during cooking. Studies have reported high amounts of Al, Pb, and Cd leaching from aluminum cookware in an acidic medium [3,10]. Odularu et al. have, however, observed aluminum leaching from different cooking pots by boiling rice in distilled water. Their study revealed that old aluminum cookware showed the highest amount of Al leaching while new steel pots had the least leaching of Al [11]. The processes of leaching can increase when the cookware is used repeatedly for a longer time. It is important to mention that new and old steel and copper cookware were not significantly different (*p* > 0.05) in terms of metal leaching.

The leaching of metals was high when preparing acidic foods rather than basic or neutral food. The leaching of aluminum and some other metals from cooking utensils provides an important source of minerals for the human body; however, their leaching in high amounts and, additionally, the toxic heavy metals present in the cookware can cause health damage. The amount of heavy metals which leach into food from cookware and utensils depends upon several factors. Highly acidic foods like tomato sauce can induce more metals to leach from cookware compared to less acidic food, like meat or chicken. Prolonged food contact, including longer storage or cooking times, may also increase the amount of the metal leaking into the food [2,9,12,13,14]. The age of the cookware and its repeated use for a long time is an additional factor that increases the intensity of metals leaching, leading to diverse negative health effects [2]. Some corrosion inhibitors are used to reduce metals leaching from cookware [3]. Curcumin aqueous extract has also been observed to reduce metals leaching effectively from aluminum cookware in solutions containing meat or vegetables at high temperatures [15].

Aluminum is a metal toxicant when inhaled in large amounts and is considered poisonous to the nervous system and brain and also causes anemia and bone diseases, especially in people suffering from kidney disease [2,16,17]. The mean exposure estimate for Al has been observed to be 125 mg per serving, more than six times the World Health Organization (WHO) Provisional Tolerable Weekly intake of 20 mg/70 kg body weight [3]. Some unpublished data from a clinical laboratory in Pakistan show a significant increase in aluminum toxicity in Pakistan, which is affecting health drastically. Lead toxicity is a serious health issue and it is attributed to difficulties such as high blood pressure, behavioral disorders, cardiovascular diseases, intellectual development, and premature death [10,18,19,20,21]. According to the World Health Organization (WHO), the admissible level of Pb is 0.01 ppm (Hussain et al., 2019). Cadmium can result in severe stomach pain, diarrhea, rampage of the respiratory tract, and kidney damage, and also causes genotoxicity [7,9,22,23]. WHO’s permissible level of Cd is 0.003 ppm [24]. Chromium is required by the human body to help facilitate insulin action in the body tissues for glucose metabolism. However, a high concentration of Cr is carcinogenic [25] and can also lead to cardiovascular, gastrointestinal, and severe respiratory disorders [23]. The admissible level of Cr allowed by WHO is 0.05 ppm [24]. Nickel is a poisonous element and causes human carcinoma, skin allergies, lung fibrosis, DNA damage, etc. [22,26]. The tolerable daily intake of Ni allowed by the WHO is 0.07 ppm [24]. The present study revealed aluminum cookware as one of the major sources of aluminum toxicity and possibly other elements, as reflected by the presence of high levels of aluminum, lead, nickel, and cadmium in the blood samples of the local population, indicating the leaching effect of food on these metals, especially from aluminum cookware. Their observed levels in serum exceeded the permissible limits, suggesting a concerning situation regarding metal contamination. These findings identify a potential source of the toxic metal contents present in the blood serum of the selected population. Numerous studies have also documented the existence of elevated levels of heavy metals in the bloodstream of individuals in Pakistan [27,28,29]. Ahmad et al., 2014; Ali et al., 2021). However, further study is required to establish a correlation between blood metal levels and metals ingested in food contaminated by cookware. The study recommends that the authorities should conduct strict surveillance on the manufacturers that make cookware to protect consumers from exposure to the risk of these toxins. Furthermore, it is imperative for end users to exercise utmost caution when utilizing aluminum cookware due to its inferior quality.

## 5. Conclusions

This study focuses on cookware as a potential contributor to metal toxicity in Pakistan. The findings of the study indicate that copper and steel cookware are relatively safe options, whereas aluminum cookware exhibits a significant risk of metal toxicity. The aluminum cookware available in Pakistan is of low quality and has an added concern of being contaminated with toxic metals, which leach into food, particularly when cooking acidic dishes. The study highlights the potential health hazards to the population due to elevated levels of aluminum and certain heavy metals, primarily resulting from leaching from cookware. Furthermore, our results shed light on the presence of higher concentrations of aluminum in blood serum compared to other heavy metals. These findings establish a basis for future investigations into the impact of utilizing low-quality cookware for food preparation on overall health and the associated burden of metal toxicity in Pakistan.

## Figures and Tables

**Table 1:** Elemental composition of various types of cookware (g/kg).

Elements	Non-Anodized Aluminum (*n* = 8)	Anodized Aluminum (*n* = 8)	Steel (*n* = 7)	Copper (*n* = 7)
Al	979.32 ± 18.19	961.34 ± 9.50	ND	ND
Fe	9.12 ± 0.54	10.72 ± 0.70	735.89 ± 15.44	8.80 ± 0.05
Pb	3.2 ± 0.25	4.64 ± 0.20	0.88 ± 0.01	2.90 ± 0.12
Cr	0.27 ± 0.008	0.20 ± 0.01	139.66 ± 3.76	ND
Ni	0.25 ± 0.01	0.38 ± 0.00	12.74 ± 0.05	2.86 ± 0.06
Cu	2.32 ± 0.07	3.67 ± 0.17	3.94 ± 0.04	887.18 ± 7.79
K	0.50 ± 0.03	0.52 ± 0.09	ND	ND
Zn	1.38 ± 0.05	2.63 ± 0.097	ND	58.49 ± 1.2
Ca	2.43 ± 0.80	3.97 ± 0.92	0.088 ± 0.001	4.26 ± 0.41
V	0.20 ± 0.06	0.05 ± 0.009	0.75 ± 0.078	ND
Mn	0.92 ± 0.01	1.43 ± 0.06	92.45 ± 5.49	ND
Sn	0.11 ± 0.03	0.149 ± 0.02	ND	9.80 ± 0.09
Si	ND	1.03 ± 0.00	13.09 ± 1.63	22.06 ± 3.26
Zr	ND	0.02 ± 0.00	ND	ND
Bi	ND	ND	0.52 ± 0.00	ND
Tb	ND	ND	ND	1.08 ± 0.70
I	ND	ND	ND	1.92 ± 0.004
S	ND	ND	ND	0.35 ± 0.07
Mo	ND	ND	ND	0.30 ± 0.004

ND = Not detected.

**Table 2:** Metals leaching (mg/L) from non-anodized and anodized aluminum cookware upon boiling with acetic acid for various time intervals.

Metals	Non-Anodized (*n* = 6 Each)				Anodized (*n* = 6 Each)			
	Time (h)				Time (h)			
	New			Old	New (*n* = 6)			Old (*n* = 6)
	0.5	1	2	1	0.5	1	2	1
Al	1116 ± 9.9	1553 ± 11.50	2144 ± 14.55	883.3 ± 6.88	151.0 ± 1.84	288.0 ± 3.40	532.1 ± 6.74	795.2 ± 7.74
Pb	1.52 ± 0.01	3.22 ± 0.02	5.66 ± 0.05	0.48 ± 0.04	1.06 ± 0.01	1.50 ± 0.06	1.95 ± 0.01	0.47 ± 0.03
Fe	5.29 ± 0.01	13.62 ± 0.05	21.62 ± 0.07	9.802 ± 0.2	1.11 ± 0.09	3.50 ± 0.02	5.61 ± 0.07	6.705 ± 0.1
Cr	0.72 ± 0.03	1.11 ± 0.04	1.38 ± 0.00	0.1 ± 0.02	0.33 ± 0.01	0.46 ± 0.00	0.76 ± 0.01	0.11 ± 0.00
Cd	0.11 ± 0.00	0.17 ± 0.01	0.30 ± 0.00	0.05 ± 0.01	0.1 ± 0.01	0.14 ± 0.01	0.22 ± 0.00	0.06 ± 0.00
Cu	1.1 ± 0.01	1.89 ± 0.004	4.15 ± 0.01	0.841 ± 0.01	0.08 ± 0.01	0.13 ± 0.04	0.18 ± 0.01	0.754 ± 0.00
Ni	0.57 ± 0.02	0.85 ± 0.02	1.44 ± 0.02	0.3 ± 0.04	0.45 ± 0.03	0.69 ± 0.02	1.0 ± 0.07	0.28 ± 0.02

**Table 3:** Metals leaching (mg/L) from stainless steel and copper cookware upon boiling with acetic acid for various time intervals.

Metals	Steel (*n* = 6 Each)				Copper (*n* = 5 Each)			
	Time (h)				Time (h)			
	New			Old	New			Old
	0.5	1	2	1	0.5	1	2	1
Al	0.411 ± 0.07	0.60 ± 0.03	0.87 ± 0.05	0.730 ± 0.23	0.02 ± 0.00	0.02 ± 0.00	0.18 ± 0.02	0.14 ± 0.06
Pb	0.03 ± 0.034	0.04 ± 0.03	0.07 ± 0.02	0.05 ± 0.01	0.47 ± 0.40	1.18 ± 0.31	1.75 ± 0.13	1.46 ± 0.02
Fe	16.22 ± 1.06	21.28 ± 1.18	51.31 ± 2.38	31.11 ± 4.01	1.32 ± 0.05	2.32 ± 0.10	4.60 ± 0.07	2.81 ± 0.04
Cr	0.38 ± 0.01	0.52 ± 0.05	2.88 ± 0.28	0.7 ± 0.01	0.01 ± 0.00	0.04 ± 0.01	0.06 ± 0.00	0.06 ± 0.01
Cd	0.01 ± 0.01	0.02 ± 0.01	0.05 ± 0.00	0.04 ± 0.00	0.05 ± 0.01	0.07 ± 0.02	0.19 ± 0.02	0.1 ± 0.00
Cu	0.19 ± 0.01	0.24 ± 0.00	0.33 ± 0.01	0.28 ± 0.00	4.05 ± 0.01	6.12 ± 0.01	10.12 ± 0.00	7.64 ± 1.01
Ni	1.02 ± 0.04	2.03 ± 0.06	3.31 ± 0.03	2.42 ± 0.05	0.00 ± 0.00	0.04 ± 0.0	0.01 ± 0.04	0.13 ± 0.09

**Table 4:** Metals leaching (mg/L) from non-anodized and anodized aluminum cookware upon boiling with sodium bicarbonate for various time intervals.

Metals	Non-Anodized (*n* = 6 Each)				Anodized (*n* = 6 Each)			
	Time (h)				Time (h)			
	New			Old	New			Old
	0.5	1	2	1	0.5	1	2	1
Al	6.10 ± 1.54	16.79 ± 1.28	72.09 ± 5.45	2.40 ± 0.21	2.62 ± 0.82	5.76 ± 0.61	9.41 ± 1.14	2.35 ± 0.27
Pb	1.37 ± 0.03	2.57 ± 0.01	3.73 ± 0.02	2.328 ± 0.27	0.26 ± 0.04	0.37 ± 0.01	0.43 ± 0.03	0.378 ± 0.04
Fe	0.65 ± 0.03	0.75 ± 0.01	1.22 ± 0.02	0.24 ± 0.01	0.58 ± 0.02	1.01 ± 0.02	2.65 ± 0.03	0.77 ± 0.06
Cr	0.19 ± 0.02	0.33 ± 0.04	0.54 ± 0.00	0.166 ± 0.01	0.05 ± 0.01	0.07 ± 0.01	0.09 ± 0.01	0.125 ± 0.01
Cd	0.01 ± 0.00	0.025 ± 0.00	0.035 ± 0.00	0.018 ± 0.01	0.00 ± 0.00	0.01 ± 0.0	0.02 ± 0.00	0.015 ± 0.00
Cu	0.14 ± 0.01	0.62 ± 0.00	1.45 ± 0.00	0.04 ± 0.00	0.37 ± 0.01	0.81 ± 0.01	1.28 ± 0.00	0.29 ± 0.01
Ni	0.29 ± 0.03	0.34 ± 0.01	0.71 ± 0.01	0.274 ± 0.05	0.07 ± 0.04	0.07 ± 0.06	0.13 ± 0.05	0.11 ± 0.06

**Table 5:** Metals leaching (mg/L) from stainless steel and copper cookware upon boiling with sodium bicarbonate for various time intervals.

Metals	Steel (*n* = 6 Each)				Copper (*n* = 5 Each)			
	Time (h)				Time (h)			
	New			Old	New			Old
	0.5	1	2	1	0.5	1	2	1
Al	0.63 ± 0.08	0.79 ± 0.06	1.08 ± 0.15	2.70 ± 0.06	0.12 ± 0.07	0.20 ± 0.16	0.41 ± 0.05	1.27 ± 0.23
Pb	0.345 ± 0.03	0.52 ± 0.01	0.85 ± 0.02	0.33 ± 0.07	0.07 ± 0.28	0.42 ± 0.22	0.78 ± 0.46	0.30 ± 0.04
Fe	1.787 ± 0.06	3.98 ± 0.02	5.86 ± 0.02	1.88 ± 0.03	0.76 ± 0.06	1.28 ± 0.07	1.62 ± 0.05	1.28 ± 0.04
Cr	0.738 ± 0.08	1.21 ± 0.04	1.76 ± 0.07	0.12 ± 0.00	0.74 ± 0.04	2.65 ± 0.13	3.33 ± 0.23	0.10 ± 0.00
Cd	0.113 ± 0.01	0.14 ± 0.01	0.19 ± 0.01	0.05 ± 0.03	0.19 ± 0.03	0.27 ± 0.01	0.47 ± 0.01	0.02 ± 0.01
Cu	0.284 ± 0.01	0.37 ± 0.01	0.48 ± 0.01	0.26 ± 0.01	0.90 ± 0.01	1.40 ± 0.01	1.90 ± 0.02	1.90 ± 0.02
Ni	0.541 ± 0.05	0.91 ± 0.06	1.34 ± 0.04	0.18 ± 0.05	0.20 ± 0.06	0.63 ± 0.07	0.98 ± 0.05	0.22 ± 0.07

**Table 6:** Metals leaching (mg/L) from non-anodized and anodized aluminum cookware upon boiling with distilled water for various time intervals.

Metals	Non-Anodized (*n* = 6 Each)				Anodized (*n* = 6 Each)			
	Time (h)				Time (h)			
	New			Old	New			Old
	0.5	1	2	1	0.5	1	2	1
Al	1.69 ± 0.03	2.27 ± 0.09	2.90 ± 0.45	1.52 ± 0.10	0.16 ± 0.02	0.60 ± 0.062	1.33 ± 0.02	1.15 ± 0.04
Pb	0.002 ± 0.00	0.003 ± 0.00	0.005 ± 0.00	0.22 ± 0.04	0.00 ± 0.00	0.001 ± 0.00	0.002 ± 0.00	0.15 ± 0.04
Fe	0.02 ± 0.00	0.04 ± 0.002	0.06 ± 0.01	0.01 ± 0.00	0.00 ± 0.00	0.01 ± 0.00	0.02 ± 0.00	0.03 ± 0.01
Cr	0.02 ± 0.04	0.04 ± 0.04	0.06 ± 0.00	0.00 ± 0.00	0.007 ± 0.00	0.02 ± 0.00	0.03 ± 0.01	0.01 ± 0.01
Cd	0.00 ± 0.00	0.004 ± 0.01	0.01 ± 0.00	0.02 ± 0.01	0.00 ± 0.00	0.001 ± 0.01	0.003 ± 0.01	0.03 ± 0.01
Cu	0.02 ± 0.00	0.03 ± 0.00	0.05 ± 0.00	0.05 ± 0.00	0.01 ± 0.00	0.02 ± 0.00	0.04 ± 0.01	0.03 ± 0.00
Ni	0.00 ± 0.00	0.02 ± 0.00	0.06 ± 0.01	0.02 ± 0.03	0.00 ± 0.00	0.004 ± 0.00	0.008 ± 0.00	0.009 ± 0.00

**Table 7:** Metals leaching (mg/L) from steel and copper cookware upon boiling with distilled water for various time intervals.

Metals	Steel (*n* = 6 Each)				Copper (*n* = 5 Each)			
	Time (h)				Time (h)			
	New			Old	New			Old
	0.5	1	2	1	0.5	1	2	1
Al	0.00 ± 0.00	0.09 ± 0.01	0.16 ± 0.01	0.05 ± 0.01	0.00 ± 0.00	0.02 ± 0.00	0.04 ± 0.00	0.005 ± 0.00
Pb	0.00 ± 0.00	0.00 ± 0.00	0.07 ± 0.00	0.02 ± 0.01	0.00 ± 0.00	0.01 ± 0.00	0.02 ± 0.00	0.02 ± 0.00
Fe	0.06 ± 0.01	0.16 ± 0.01	0.39 ± 0.04	0.30 ± 0.03	0.08 ± 0.04	0.1 ± 0.010	0.2 ± 0.05	0.00 ± 0.00
Cr	0.05 ± 0.03	0.14 ± 0.02	0.24 ± 0.02	0.03 ± 0.01	0.00 ± 0.00	0.00 ± 0.00	0.005 ± 0.00	0.01 ± 0.00
Cd	0.00 ± 0.01	0.02 ± 0.00	0.03 ± 0.00	0.03 ± 0.01	0.04 ± 0.01	0.07 ± 0.01	0.09 ± 0.00	0.03 ± 0.01
Cu	0.01 ± 0.00	0.01 ± 0.01	0.16 ± 0.03	0.00 ± 0.00	0.03 ± 0.00	0.04 ± 0.01	0.06 ± 0.02	0.01 ± 0.00
Ni	0.00 ± 0.00	0.00 ± 0.0	0.004 ± 0.00	0.08 ± 0.03	0.00 ± 0.00	0.00 ± 0.00	0.006 ± 0.00	0.009 ± 0.03

**Table 8:** Total metals (mg/L) leached from various cookware under acidic, basic, and neutral conditions.

Acetic Acid						Sodium Bicarbonate				Distilled Water		
		Time (h)				Time (h)				Time (h)		
Types of Condition		New		Old		New		Old		New		Old
	0.5	1	2	1	0.5	1	2	1	0.5	1	2	1
Non- anodized	1125.31 ± 9.92 ^a^	1573.86 ± 11.52 ^a^	2178.55 ± 14.57 ^a^	894.87 ± 6.92 ^a^	8.75 ± 1.66 ^a^	21.425 ± 1.34 ^a^	79.78 ± 5.50 ^a^	5.47 ± 0.56 ^a^	1.75 ± 0.07 ^a^	2.41 ± 0.13 ^a^	3.145 ± 0.47 ^a^	1.84 ± 0.18 ^a^
Anodized	154.13 ± 1.87 ^b^	294.42 ± 3.42 ^b^	541.42 ± 6.81 ^b^	803.58 ± 7.76 ^b^	3.95 ± 0.84 ^b^	8.10 ± 0.72 ^b^	14.01 ± 1.26 ^b^	4.038 ± 0.45 ^a^	0.177 ± 0.02 ^b^	0.66 ± 0.072 ^b^	1.43 ± 0.05 ^b^	1.41 ± 0.11 ^a^
Stainless steel	18.261 ± 1.23 ^c^	24.73 ± 1.36 ^c^	58.82 ± 2.77 ^c^	35.33 ± 4.31 ^c^	4.44 ± 0.32 ^b^	7.92 ± 0.21 ^b^	11.56 ± 0.32 ^c^	5.52 ± 0.25 ^a^	0.12 ± 0.05 ^b^	0.42 ± 0.05 ^b^	1.054 ± 0.1 ^b^	0.51 ± 0.1 ^b^
Copper	5.92 ± 0.47 ^d^	9.79 ± 0.45 ^d^	16.91 ± 0.28 ^d^	12.34 ± 1.23 ^d^	2.98 ± 0.55 ^c^	6.85 ± 0.67 ^c^	9.49 ± 0.87 ^d^	5.09 ± 0.41 ^a^	0.15 ± 0.05 ^b^	0.24 ± 0.03 ^c^	0.421 ± 0.07 ^c^	0.084 ± 0.04 ^c^

Mean values with different lowercase letters in the same columns for the same time point are significantly different (*p* < 0.05), ordered according to a > b > c > d.

**Table 9:** Metals leaching (mg/L) from new non-anodized and anodized aluminum cookware during cooking meat for 1 h.

Concentration (ppm)				
Metals	Non-Anodized (*n* = 4 Each)		Anodized (*n* = 4 Each)	
	New	Old	New	Old
Al	243.6 ± 5.16	181 ± 7.23	112.05 ± 4.23	165.05 ± 4.90
Pb	1.20 ± 0.04	0.80 ± 0.04	0.4 ± 0.07	0.7 ± 0.10
Cr	0.43 ± 0.01	0.30 ± 0.05	0.33 ± 0.01	0.41 ± 0.06
Cd	0.06 ± 0.00	0.04 ± 0.00	0.003 ± 0.00	0.02 ± 0.01
Ni	0.34 ± 0.02	0.24 ± 0.01	0.007 ± 0.01	0.01 ± 0.01
Total	245.29 ± 5.23 ^a^	182.45 ± 7.33 ^b^	112.79 ± 4.32 ^d^	166.19 ± 5.08 ^c^

*p* > 0.05 a vs. b; *p* > 0.05 c vs. d; *p* > 0.05 a vs. d; *p* > 0.05 b vs. c, where a > b > c > d.

**Table 10:** The concentrations of metals in the blood serum of the local population.

Metals	Concentration (mg/L)
Al	13.34 ± 0.49
Pb	1.32 ± 0.19
Cd	0.51 ± 0.016
Ni	2.17 ± 0.10

## Data Availability

Data will be available on request to the corresponding author.
